# Frailty index of deficit accumulation and falls: data from the Global Longitudinal Study of Osteoporosis in Women (GLOW) Hamilton cohort

**DOI:** 10.1186/1471-2474-15-185

**Published:** 2014-05-29

**Authors:** Guowei Li, George Ioannidis, Laura Pickard, Courtney Kennedy, Alexandra Papaioannou, Lehana Thabane, Jonathan D Adachi

**Affiliations:** 1 Department of Clinical Epidemiology & Biostatistics, McMaster University, 1280 Main Street West, Hamilton, ON L8S 4 L8, Canada; 2 Department of Medicine, McMaster University, 1280 Main Street West, Hamilton, ON L8S 4 L8, Canada; 3 St. Joseph’s Hospital, McMaster University, 25 Charlton Avenue East, Hamilton, ON L8N 1Y2, Canada

**Keywords:** Frailty, Falls, Fracture, Death, Hospitalization

## Abstract

**Background:**

To investigate the association between frailty index (FI) of deficit accumulation and risk of falls, fractures, death and overnight hospitalizations in women aged 55 years and older.

**Methods:**

The data were from the Global Longitudinal Study of Osteoporosis in Women (GLOW) Hamilton Cohort. In this 3-year longitudinal, observational cohort study, women (N = 3,985) aged ≥55 years were enrolled between May 2008 and March 2009 in Hamilton, Canada. A FI including co-morbidities, activities of daily living, symptoms and signs, and healthcare utilization was constructed using 34 health deficits at baseline. Relationship between the FI and falls, fractures, death and overnight hospitalizations was examined.

**Results:**

The FI was significantly associated with age, with a mean rate of deficit accumulation across baseline age of 0.004 or 0.021 (on a log scale) per year. During the third year of follow-up, 1,068 (31.89%) women reported at least one fall. Each increment of 0.01 on the FI was associated with a significantly increased risk of falls during the third year of follow-up (odds ratio [OR]: 1.02, 95% confidence interval [CI]: 1.02-1.03). The area under the curve (AUC) of the predictive model was 0.69 (95% CI: 0.67-0.71). Results of subgroup and sensitivity analyses indicated the relationship between the FI and risk of falls was robust, while bootstrap analysis judged its internal validation. The FI was significantly related to fractures (hazard ratio [HR]: 1.02, 95% CI: 1.01-1.03), death (OR: 1.05, 95% CI: 1.03-1.06) during the 3-year follow-up period and overnight hospitalizations (incidence rate ratio [IRR]: 1.02, 95% CI: 1.02-1.03) for an increase of 0.01 on the FI during the third year of follow-up. Measured by per standard deviation (SD) increment of the FI, the ORs were 1.21 and 1.40 for falls and death respectively, while the HR was 1.17 for fractures and the IRR was 1.18 for overnight hospitalizations respectively.

**Conclusion:**

The FI of deficit accumulation increased with chronological age significantly. The FI was associated with and predicted increased risk of falls, fractures, death and overnight hospitalizations significantly.

## Background

Frailty is a state of vulnerability and is highly associated with age [1,2]. Frailty has been described as: ‘Frailty is a dynamic state affecting an individual who experiences losses in one or more domains of human functioning (physical, psychological, social) that are caused by the influence of a range of variables and which increases the risk of adverse outcomes’ [3]. Frailty is increasing in our aging population, with an overall prevalence of 10.7% in community-dwellers aged 65 years and older as reported in a systematic review [4]. Furthermore, it is estimated that 25-50% of adults ≥85 years are frail [2]. Frailty is strongly related to an increased risk of adverse outcomes, including institutionalization, disability and death [2,5].

Ideally, frailty models should have both discriminative and evaluative properties, and should reliably reflect the natural history of aging [2]. The phenotypic model [6] and cumulative deficit model [7] are the predominant models currently used. Both have been validated by several studies [2]. However, even with overlap and statistical convergence, the continuous frailty index (FI) of deficit accumulation presents greater discriminatory ability for people with frailty than that of the categorical phenotypic model [8,9]. Application of a FI may be more accurate to help identify the frail elderly for further preventive interventions [2,10]. Unfortunately, no practical widely-accepted FI exists and a more efficient model to detect and measure frailty is urgently needed in routine clinical practice [1,2].

Falls are a very important public health issue for the elderly and will increase in frequency as the proportion of elderly grows larger [11]. One third of community-dwellers aged >65 years have at least one fall each year, often resulting in pain syndromes, functional limitations, dislocations, serious soft tissue injuries, fractures, immense health-care costs and high mortality [11,12]. E.g., up to 12% of all falls in the elderly are followed by a fracture, and 23% of trauma-related deaths in patients >65 years and 34% in those >85 years follow a fall [11,13]. The relationship among frailty and falls has been investigated in some studies based on the frailty phenotypic model and its variants [14-17], but few have studied the relationship using the continuous FI. More evidence regarding the relationship between frailty and falls with the use of a FI will be helpful at a clinical research level and at a health care policy level [7]. Furthermore, since previous studies typically only reported their findings on participants aged ≥65 years [17], studies investigating the relationship between frailty and adverse outcomes in adults younger than 65 years are needed.

In this study, using the Global Longitudinal Study of Osteoporosis in Women (GLOW) 3-year Hamilton cohort, our primary objective was to examine the association between frailty and the risk of falls in women aged 55 years and older, by constructing a feasible and pragmatic FI. The secondary objective was to investigate the relationship between frailty and risk of fractures, death and overnight hospitalizations. We hypothesized that a higher FI was associated with increased risk of falls, fractures, death and overnight hospitalizations significantly.

## Methods

### Participants and setting

The Global Longitudinal Study of Osteoporosis in Women (GLOW) is an observational study designed to explore risk factors for and health consequences of fragility fractures in 60,393 women aged 55 years and older who had consulted their physician in the past 24 months, involving 723 physician practices at 17 sites in 10 countries (Australia, Belgium, Canada, France, Germany, Italy, Netherlands, Spain, UK, and US). Each site obtained local ethics committee approval to participate in the study. This has been described in detail previously [18]. Briefly, based on the GLOW Hamilton cohort, a sample of approximately 4,000 participants were enrolled between May 2008 and March 2009 and stratified according to age strata such that two-thirds of participants were aged no less than 65 years. Women were eligible for inclusion if they had no language barriers or cognitive impairment, and were not too ill or institutionalized to complete the study survey [18].

Participants were surveyed annually with mailed questionnaires. Telephone interviews were performed if the participant needed assistance with finishing the survey or did not return mailed questionnaires. Surveys used in the GLOW study were designed to be self-administered by participants and covered the domains as follows: participant characteristics and risk factors, perception about fracture risk and osteoporosis, medication use, co-morbidities, health care use and access, physical activity, physical function and quality of life [18]. This study only used data in the Hamilton site, Canada, while the other 16 sites were not involved in this study. Specifically, our study was a longitudinal analysis of the 3-year GLOW cohort of women in Hamilton. Written informed consent was obtained from all participants, and the study was reviewed and approved by the Western Institutional Review Board.

### Construction of the FI at baseline

We constructed our FI based on the standard procedure and framework suggested by Searle and Rockwood [1,19]. In creating a FI, the selection of variables should satisfy four basic criteria: biologically sensible, accumulate with age, do not saturate too early, and collectively cover a range of systems [19]. In previous studies, 30 to 70 deficits had been used to construct the FI [1,20]. The deficit accumulation approach does not necessarily require the exact same variables or the same number of deficits, to form the FI [20,21]. Searle, however, recommended that the FI should include at least 30 to 40 total deficits [19]. The finalized FI consisted of 34 variables at baseline, which included co-morbidities (n = 15), activities of daily living (ADL) (n = 12), symptoms and signs (n = 6), and healthcare utilization (n = 1). However, no measure of on social support deficits could be included in the FI because no such variable was recorded in the GLOW study. Regarding each variable, the consensus on its eligibility among authors was reached before it was included to construct the FI. As suggested by Rockwood and Searle [7,19], each deficit variable was dichotomized or polychotomized and mapped to the interval 0–1 (e.g., for self-rating of health, ‘Excellent’ was coded as 0, ‘very good’ as 0.25, ‘good’ as 0.5, ‘fair’ as 0.75 and ‘poor’ as 1) to represent the frequency or severity of the deficit. None of the included deficit variables had more than 5% missing values.

The FI was calculated by adding up the values of deficits and dividing by the total number of items (n = 34), with the FI ranging from 0 to 1. For instance, if an individual had three deficits with each score of 1 point, two deficits with each score of 0.5 point and the other 29 deficits with each score of 0, her cumulative values of deficits would therefore be 4 divided by 34 giving a FI = 0.12.

### Outcomes

The outcomes included falls, fractures, death and overnight hospitalizations. All the outcomes were self-reported and information from medical records was not available.

The primary outcome in this study, falls, were measured at baseline and each year of follow-up. Participants reported number of incident falls (none, one time, more than one time) in the prior 12 months on the annually mailed questionnaires.

Women were identified as having baseline self-reported fractures that included fractures of the clavicle, upper arm, wrist, spine, rib, hip, pelvis, ankle, upper leg or lower leg since the age of 45 years. Incident fractures and the dates of the fractures were reported on the 1-, 2- and 3- year follow-up surveys. At baseline, participants categorized their number of overnight hospitalizations according to the options on the questionnaire (none, one time, two times, more than two times). At each follow-up year, they were asked to record the total number of hospitalizations and total number of nights spent in hospital. Death was ascertained through contact with participants’ spouses, friends or family members and through electronic searches of obituaries. Some spouses and family members notified us of the participant’s death when they received mailings from our office, or when we called the homes of those participants who did not mail back their annual questionnaire. If we were unable to contact the household of the non-responders, we searched electronic databases of obituaries for entries which matched the participant’s full names and dates of birth.

### Statistical analyses

The continuous FI was reported as mean and standard deviation (SD). Comparison of the categorized falls status (none, one time, more than one time) at baseline was examined using Chi-square tests for categorical variables and analysis of variance (ANOVA) for continuous variables. The rate of deficit accumulation per year at baseline was calculated on the basis of the mean FI with age. To make the results comparable with other studies [1,19,20], the rate of deficit accumulation was reported on a crude scale as well as on a log scale.

The relationship between each individual variable included in the FI and risk of falls during the third year of follow-up was also examined, after adjusted for baseline age. Binary logistic regression (i.e., had falls versus no falls) was performed to assess the association between baseline FI and risk of falls if the proportional odds assumption for ordinal logistic regression (i.e., no falls, one fall, more than one fall) was not met, taking women with no falls as the reference category.

Because the dates for falls were not available, unless otherwise emphasized, analyses of the relationship between baseline FI and risk of falls was conducted only using the data on the falls during the third year of follow-up. Baseline age-adjusted binary logistic regression models and fully-adjusted multivariable logistic regression models were performed and compared, where fully-adjusted models were adjusted for age, body mass index (BMI), smoking, drinking, education and baseline falls, to analyze the association between baseline FI and risk of falls. Receiver operating characteristic curves (ROC) were used to calculate the areas under the curve (AUC), which could judge the discriminability of the FI. A bootstrap analysis resampling 1,000 times with replacement from the original sample was conducted to assess the internal validation of the relationship between the FI and falls [22]. Moreover, a sensitivity analysis was performed to investigate the relationship between the FI and the incident falls during the 3-year follow-up period, in which participants were dichotomized as having new incident falls (i.e., without baseline falls) and having recurrent falls (i.e., with baseline falls).

Secondary outcomes were fractures, death and overnight hospitalization. Similarly, age-adjusted models and fully-adjusted models were carried out and compared. Because the dates for death and overnight hospitalization were unavailable, logistic regression was applied to death during the follow-up, while Poisson regression was used to analyze the relationship between the FI and overnight hospitalizations during the third year of follow-up given the number of overall nights spent in the hospital as count data. Cox proportional hazards regression based on time-to-event was used to assess the associations between the FI and fractures after adjusting for age, BMI, smoking, drinking, education, baseline fractures and family history of fractures, where both a statistical test of proportional hazards assumption and a graphical examination using Schoenfeld residuals were performed [23].

To compare the results with other studies’ findings [8,19,24], all the statistics on the associations between the FI and falls, fractures, death and overnight hospitalization were reported based on an increase of 0.01 on the FI. The statistics were also measured and presented by per 1-SD increment of the FI.

Given that the findings on participants aged <65 years were scanty [17], subgroup analyses were conducted using the cut-off point of 65 years for age. For missing data, if <10% of observations on a variable were missing, the mean or median of the variable in its group was imputed [25]. If ≥10% of data were missing, assuming they were missing at random, we conducted multiple imputations by including other relevant variables that were judged by clinical knowledge [26,27]. All statistical tests were two-sided using an alpha level of 0.05 and all analyses were conducted with the software package SAS, version 9.3 (SAS Institute, Inc., Cary, NC).

## Results

### Characteristics of participants at baseline

Characteristics of the GLOW Hamilton cohort are presented in Table 1. A total of 3,985 women provided information at baseline and were included for analyses. The mean age was 69.4 (SD: 8.89) years, and about one third of the women (35%) were younger than 65 years old. Their mean body mass index (BMI) was 27.7 (SD: 5.77) kg/m^2^. Approximately 11% of the women were smokers and 49% drank alcohol. Most participants did not have a family history of fractures (76%), overnight hospitalizations (89%) in the past year, or fractures since 45 years old (78%).

**Table 1:** Baseline characteristics of study participants*

**Characteristics**	**Overall participants (n = 3,985)**	**Women without falls (n = 2471)**	**Women with one fall (n = 853)**	**Women with ≥2 falls (n = 630)**	**P-value**
Age: mean (SD^a^), years	69.4 (8.89)	69.4 (8.58)	69.6 (9.14)	69.4 (9.74)	0.748^c^
Age strata, n (%)					
55-64	1,385 (34.76)	839 (33.95)	292 (34.23)	248 (39.37)	0.404^d^
65-74	1,423 (35.71)	939 (38.00)	285 (33.41)	187 (29.68)	
75-84	952 (23.89)	578 (23.39)	219 (25.67)	142 (22.54)	
≥85	225 (5.65)	115 (4.65)	57 (6.68)	53 (8.41)	
BMI^b^: mean (SD), kg/m^2^	27.7 (5.77)	27.5 (5.54)	27.7 (5.90)	28.5 (6.40)	0.002^c^
Smoker, n (%)					
Yes	447 (11.3)	269 (10.96)	97 (11.44)	77 (12.32)	0.623^e^
No	3,510 (88.70)	2185 (89.04)	751 (88.56)	548 (87.68)	
Drinking (drinks/week), n (%)					
0	2,027 (51.21)	1239 (50.49)	411 (48.52)	360 (57.51)	0.057^d^
<7	1,414 (35.73)	886 (36.10)	324 (38.25)	193 (30.83)	
7-13	428 (10.81)	276 (11.25)	94 (11.10)	56 (8.95)	
≥14	89 (2.25)	53 (2.16)	18 (2.13)	17 (2.72)	
Race, n (%)					
White	3,717 (93.27)	2299 (93.04)	798 (93.55)	597 (94.76)	0.293^e^
Non-white	268 (6.73)	172 (6.96)	55 (6.45)	33 (5.24)	
Education, n (%)					
High school or less	2,509 (64.10)	1608 (66.23)	518 (61.74)	360 (58.16)	<0.001^e^
More than high school	1,405 (35.90)	820 (33.77)	321 (38.26)	259 (41.84)	
Family history of fractures, n (%)					
Yes	898 (23.81)	514 (21.82)	204 (25.40)	172 (29.30)	<0.001^e^
No	2,874 (76.19)	1842 (78.18)	599 (74.60)	415 (70.70)	
Overnight hospitalization in last 12 months, n (%)					
0	3,498 (88.65)	2213 (90.25)	747 (88.30)	516 (82.96)	<0.001^d^
1	337 (8.54)	187 (7.63)	78 (9.22)	69 (11.09)	
≥2	111 (2.81)	52 (2.12)	21 (2.48)	37 (5.95)	
Prior fractures since 45 years old, n (%)					
Yes	862 (22.31)	447 (18.65)	217 (26.08)	193 (31.64)	<0.001^e^
No	3,001 (77.69)	1950 (81.35)	615 (73.92)	417 (68.36)	
Frailty index: mean (SD)	0.24 (0.13)	0.22 (0.12)	0.25 (0.13)	0.31 (0.15)	<0.001^c^

*Mean follow-up = 3.01 years.

^a^SD: standard deviation; ^b^BMI: body mass index; ^c^One-way analysis of variance (ANOVA) test; ^d^Mantel-Haenszel Chi-square test; ^e^Chi-square test.

There were 62% (n = 2,471), 22% (n = 853), 16% (n = 630) of the women who reported no falls, one fall and no less than two falls at baseline, respectively. With the increased number of falls, participants tended to be more educated (P < 0.001) and with higher BMI (P = 0.002). More family history of fractures, overnight hospitalizations and prior fractures were reported as the number of baseline falls increased (P < 0.001). However, no differences in age, ethnicity, smoking and drinking status were found between the three groups (Table 1).

### FI and its relation to age

**Figure 1:**
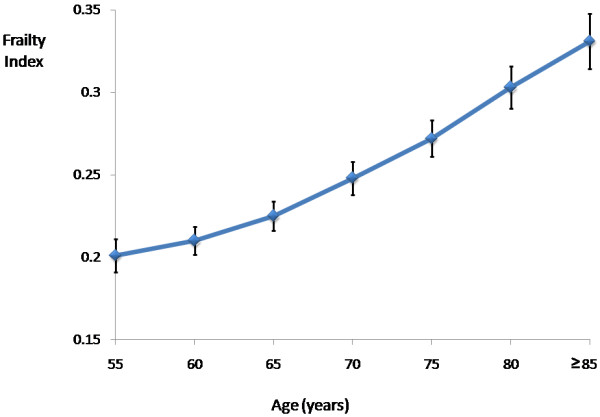
The frailty index with 95% confidence intervals grouped according to 5-year intervals from age 55.

Thirty-four variables were included in constructing the FI. Each individual deficit variable, their coding and their relationship with falls at year 3 of follow-up are shown in Table 2. Most deficits demonstrated significant associations with increased risk of falls using logistic regression analyses adjusted for baseline age.The mean FI was 0.24 (SD: 0.13) for all the women, and the 99% upper limit of the FI was 0.59. The means of the FI were 0.21 (SD: 0.12) and 0.26 (SD: 0.13) for women aged <65 and ≥65 years old, respectively. FI was positively correlated with age (r = 0.29, P < 0.001). Considering deficits cumulatively, the mean FI increased with age (Figure 1). The mean rate of deficit accumulation across ages at baseline per year was 0.004 (95% confidence interval [CI]: 0.004-0.005), or 0.021 (95% CI: 0.019-0.024) on a log scale. However, the mean rate of the FI was not significant for younger women (β = 0.002, 95% CI: −0.001-0.004; β = 0.014, 95% CI: −0.001-0.030 on a log scale), whereas higher FI was significantly associated with increased age for women aged ≥65 years (β = 0.005, 95% CI: 0.005-0.006; β = 0.024, 95% CI: 0.020-0.027 on a log scale).

**Table 2:** Coding of individual variables in the frailty index and their odds ratios of falls during the third year of follow-up

**Variables**	**Coding**	**Falls**	
		**OR (95% CI, P-value)**^**a**^	**n (%)**^**b**^
***Co-morbidities (n = 15)***			
Taking/taken five or more medications^c^	Yes = 1, no = 0	1.30 (1.07-1.59) 0.008	607 (15.23)
Has chronic bronchitis or emphysema^c^	Yes = 1, no = 0	1.40 (1.08-1.81) 0.012	307 (7.93)
Has osteoarthritis or degenerative joint disease^c^	Yes = 1, no = 0	1.53 (1.32-1.78) <0.001	1,354 (35.05)
Has rheumatoid arthritis^c^	Yes = 1, no = 0	1.45 (1.16-1.81) <0.001	447 (11.57)
Suffers from stroke^c^	Yes = 1, no = 0	2.16 (1.55-3.00) <0.001	193 (4.92)
Has ulcerative colitis or Crohn’s disease^c^	Yes = 1, no = 0	2.08 (1.32-3.28) 0.002	82 (2.09)
Has celiac disease^c^	Yes = 1, no = 0	1.20 (0.50-2.88) 0.683	25 (0.64)
Has Parkinson’s disease^c^	Yes = 1, no = 0	3.09 (0.98-9.74) 0.054	18 (0.46)
Has multiple sclerosis^c^	Yes = 1, no = 0	1.28 (0.52-3.17) 0.596	22 (0.56)
Has cancer^c^	Yes = 1, no = 0	1.06 (0.86-1.32) 0.570	502 (12.77)
Has diabetes (type-1)^c^	Yes = 1, no = 0	1.44 (1.00-2.08) 0.053	165 (4.22)
Has hypertension^c^	Yes = 1, no = 0	1.14 (0.99-1.32) 0.077	2,025 (51.63)
Has heart disease^c^	Yes = 1, no = 0	1.52 (1.25-1.86) <0.001	623 (16.01)
Has high cholesterol^c^	Yes = 1, no = 0	1.15 (1.00-1.33) 0.050	1,752 (45.03)
Self rating of health^d^	Excellent = 0, very good = 0.25, good = 0.5, fair = 0.75, poor = 1	2.77 (1.99-3.86) <0.001	73 (1.85)
***Activities of daily living (ADL) (n = 12)***			
Limitations in vigorous activities^c^	Yes = 1, a little = 0.5, no = 0	1.67 (1.37-2.05) <0.001	1,735 (45.49)
Limitations in moderate activities^d^	Yes = 1, a little = 0.5, no = 0	2.04 (1.65-2.52) <0.001	569 (14.59)
Limitations in lifting or carrying groceries^d^	Yes = 1, a little = 0.5, no = 0	2.01 (1.61-2.52) <0.001	416 (10.62)
Limitations in climbing one flight of stairs^d^	Yes = 1, a little = 0.5, no = 0	2.22 (1.76-2.80) <0.001	374 (9.69)
Limitations bending, kneeling or stooping^d^	Yes = 1, a little = 0.5, no = 0	2.02 (1.66-2.46) <0.001	781 (20.12)
Limitations walking one hundred yards^d^	Yes = 1, a little = 0.5, no = 0	1.97 (1.56-2.48) <0.001	378 (9.88)
Limitations in bathing or dressing yourself^d^	Yes = 1, a little = 0.5, no = 0	1.88 (1.37-2.59) <0.001	154 (3.93)
Needs arms to help stand up from a chair^d^	Yes = 1, no = 0	1.83 (1.57-2.13) <0.001	1,495 (37.98)
Number of days to walk at least 20 minutes in the past 30 days^d^	≤2 days = 1, >2 days = 0	1.21 (1.02-1.43) 0.025	1,081 (27.50)
Self rating of mobility^d^	Unable = 1, some problem = 0.5, no problem = 0	3.05 (2.22-4.18) <0.001	1 (0.03)
Self rating of self-care^d^	Unable = 1, some problem = 0.5, no problem = 0	6.83 (3.75-12.43) <0.001	4 (0.10)
Self rating of usual activities^d^	Unable = 1, some problem = 0.5, no problem = 0	3.60 (2.68-4.84) <0.001	56 (1.41)
***Symptoms and sig*** **ns (n = 6)**			
Feels full of life^d^	All the time = 0, most of time = 0.25, some time = 0.5, a little time = 0.75, none of time = 1	3.61 (2.66-4.89) <0.001	228 (5.84)
Has a lot of energy^d^	All the time = 0, most of time = 0.25, some time = 0.5, a little time = 0.75, none of time = 1	3.91 (2.88-5.31) <0.001	263 (6.73)
Feels worn out^d^	All the time = 1, most of time = 0.75, some time = 0.5, a little time = 0.25, none of time = 0	3.73 (2.73-5.09) <0.001	123 (3.15)
Feels tired^d^	All the time = 1, most of time = 0.75, some time = 0.5, a little time = 0.25, none of time = 0	4.14 (2.98-5.76) <0.001	173 (4.40)
Self rating of pain/discomfort^d^	Extremely = 1, moderate = 0.5, no = 0	3.22 (2.43-4.28) <0.001	242 (6.15)
Unintentional weight loss of 10 pounds^c^	Yes = 1, no = 0	1.42 (1.12-1.80) 0.004	433 (11.01)
***Healthcare utilization *** **(n = 1)**			
Times of visiting a healthcare provider to get medical care in the past year^c^	None = 0, 1–2 times = 0.33, 3–5 times = 0.67, 6 or more times = 1	1.39 (1.28-1.51) <0.001	842 (21.43)

^a^OR: odds ratio, adjusted for baseline age; CI: confidence interval.

^b^For participants whose score was 1 in individual variable.

^c^Ordinal logistic regression results, p-values of tests for the proportional odds assumption were >0.05.

^d^Binary logistic regression since proportional odds assumption was not met.

### Relationship between the FI and falls

During the third year of follow-up, 32% (n = 1,068) reported at least one fall in the past year (Table 3). Results of fully-adjusted multivariable logistic regression showed that the relationship between the FI and falls was significant (odds ratio [OR]: 1.02, 95% CI: 1.02-1.03 for an increase of 0.01 on the FI; OR: 1.21, 95% CI: 1.14-1.27 for an increase of per SD). The FI predicted falls during the third year of follow-up with an AUC of 0.69 (95% CI: 0.67-0.71). When a cut-off point of 65 years was applied, 32% reported falls for both age strata (Table 3). Nevertheless, the OR was higher, though not significantly, for older women (OR = 1.03) than for younger women aged <65 years (OR = 1.02). Similar results were also found in the age-adjusted models.

**Table 3:** Relationship between frailty index and falls, fractures, death and overnight hospitalizations during the third year of follow-up or during the 3-year follow-up for the whole group and age subgroups

**Outcomes**	**n (%)**	**Age-adjusted models**		**Fully-adjusted models**	
		**Statistics (95% CI)**^**a**^	**P-value**	**Statistics (95% CI)**^**a**^	**P-value**
**Falls**^**b**^	1,068 (31.89)	1.03 (1.03-1.04)	<0.001	1.02 (1.02-1.03)	<0.001
Women < 65 years	395 (31.98)	1.03 (1.02-1.04)	<0.001	1.02 (1.01-1.03)	0.001
Women ≥ 65 years	673 (31.84)	1.03 (1.02-1.04)	<0.001	1.03 (1.02-1.04)	<0.001
**Fractures**^**c**^					
*All fractures*^*d*^	238 (6.36)	1.02 (1.01-1.03)	<0.001	1.02 (1.01-1.03)	<0.001
Women < 65 years	71 (5.35)	1.02 (1.002-1.04)	0.032	1.02 (1.00-1.04)	0.019
Women ≥ 65 years	167 (6.92)	1.02 (1.01-1.03)	0.002	1.02 (1.01-1.03)	0.002
*Hip fracture*	18 (0.51)^g^	1.04 (1.00-1.07)	0.041	1.04 (1.00-1.07)	0.051
Women < 65 years	4 (0.30)	—^k^	—^k^	—^k^	—^k^
Women ≥ 65 years	14 (0.58)	1.03 (0.99-1.07)	0.201	1.02 (0.99-1.07)	0.230
*Spine fracture*	28 (0.79)^h^	1.04 (1.01-1.07)	0.004	1.03 (1.00-1.06)	0.024
Women < 65 years	6 (0.45)	—^k^	—^k^	—^k^	—^k^
Women ≥ 65 years	22 (0.91)	1.04 (1.01-1.07)	0.009	1.03 (1.00-1.06)	0.083
*Other fractures*	208 (5.56)^i^	1.02 (1.01-1.03)	<0.001	1.02 (1.01-1.03)	<0.001
Women < 65 years	65 (4.90)	1.02 (1.00-1.04)	0.021	1.02 (1.00-1.05)	0.027
Women ≥ 65 years	143 (5.92)	1.02 (1.00-1.03)	0.008	1.02 (1.00-1.03)	0.015
**Death**^**e**^	107 (2.69)	1.04 (1.03-1.06)	<0.001	1.05 (1.03-1.06)	<0.001
Women < 65 years	12 (0.87)	1.04 (0.99-1.08)	0.093	1.05 (1.01-1.10)	0.028
Women ≥ 65 years	95 (3.65)	1.05 (1.03-1.06)	<0.001	1.05 (1.03-1.06)	<0.001
**Overnight hospitalizations**^**f**^	347 (8.75)^j^	1.03 (1.02-1.04)	<0.001	1.02 (1.02-1.03)	<0.001
Women < 65 years	75 (5.43)^j^	1.07 (1.06-1.08)	<0.001	1.05 (1.04-1.06)	<0.001
Women ≥ 65 years	272 (10.52)^j^	1.02 (1.01-1.03)	<0.001	1.02 (1.01-1.02)	<0.001

^a^Statistics included odds ratio (OR) for falls and death, hazard ratio (HR) for fractures, and incidence rate ratio (IRR) for overnight hospitalization; CI: confidence interval; All statistics measured a change of 0.01 on the frailty index.

^b^Areas under the receiver operating characteristic curves (AUC) are 0.61 for age-adjusted model and 0.69 for fully-adjusted model respectively; Fully-adjusted models used binary logistic regression, adjusted for age, BMI, smoking, drinking, education, and baseline falls.

^c^Cox hazards regression; Fully-adjusted models were adjusted for age, BMI, smoking, drinking, education, baseline fracture and family history of fractures.

^d^Included hip, spine, pelvis, ribs, clavicle, wrist, upper arm, ankle, upper leg and lower leg fractures.

^e^AUC are 0.79 and 0.80 for age-adjusted model and fully-adjusted model respectively; Fully-adjusted models used binary logistic regression, adjusted for age, BMI, smoking, drinking, and education.

^f^Poisson regression; Fully-adjusted models were adjusted for age, BMI, smoking, drinking, education, and baseline overnight hospitalization.

^g^Analysis excluded women with nonhip fractures.

^h^Analysis excluded women with nonspine fractures.

^i^Analysis excluded women with hip and spine fractures.

^j^For participants spent at least one night in hospital during the third year of follow-up.

^k^Analyses could not be conducted because of the limited sample size.

Table 4 shows results of sensitivity analyses of the relationship between the FI and falls. There were 1,483 women reporting falls at baseline, and each increment of 0.01 on the FI was related to a significant 3.6% increased risk of falls (P < 0.001) in the fully-adjusted model. During the follow-up, 2,339 women reported at least one fall, among whom 1,204 had new incident falls (i.e., without baseline falls) and 1,135 reported recurrent falls (i.e., with baseline falls). The FI was associated with the increased risk of new incident and recurrent falls, with an OR of 1.02 and 1.04 respectively. Since 16% of participants (n = 636) had missing falls data during the third year of follow-up, 10 multiple imputations were conducted to estimate the association between the FI and falls. The imputed result (OR: 1.02, 95% CI: 1.02-1.03) was very similar to that without imputations (OR: 1.02, Table 3). Furthermore, results of age-adjusted models and subgroup analyses by age-stratum were consistent with the analyses in the fully-adjusted models (Table 4).

**Table 4:** Sensitivity analysis of relationship between frailty index and falls for the whole group and age subgroups

**Sensitivity analysis**	**n (%)**	**Age-adjusted models**			**Fully-adjusted models**		
		**OR (95% CI)**^**a**^	**P-value**	**AUC**^**b**^	**OR (95% CI)**	**P-value**	**AUC**
**Any falls at baseline and during the 3-year follow-up**^**c**^							
*Baseline falls*	1483 (37.51)	1.03 (1.03-1.04)	<0.001	0.61	1.04 (1.03-1.04)	<0.001	0.62
Women < 65 years	540 (39.16)	1.04 (1.03-1.05)	<0.001	0.64	1.04 (1.03-1.05)	<0.001	0.64
Women ≥ 65 years	943 (36.62)	1.03 (1.02-1.04)	<0.001	0.61	1.03 (1.03-1.04)	<0.001	0.62
*New incident falls*^*d*^	1204 (51.43)	1.02 (1.02-1.03)	<0.001	0.59	1.02 (1.02-1.03)	<0.001	0.60
Women < 65 years	384 (47.64)	1.02 (1.00-1.03)	0.015	0.56	1.02 (1.00-1.03)	0.018	0.58
Women ≥ 65 years	820 (53.42)	1.02 (1.02-1.03)	<0.001	0.61	1.03 (1.02-1.04)	<0.001	0.61
*Recurrence of falls*^*e*^	1135 (81.54)	1.04 (1.03-1.05)	<0.001	0.64	1.04 (1.03-1.06)	<0.001	0.64
Women < 65 years	419 (80.58)	1.05 (1.03-1.07)	<0.001	0.66	1.06 (1.03-1.08)	<0.001	0.68
Women ≥ 65 years	716 (82.11)	1.04 (1.02-1.05)	<0.001	0.63	1.04 (1.02-1.05)	<0.001	0.66
**Analysis with multiple imputation for missing data**^**f**^	1317 (33.05)^g^	1.03 (1.03-1.04)	<0.001	0.61	1.02 (1.02-1.03)	<0.001	0.69
Women < 65 years	448 (32.35)^g^	1.03 (1.02-1.04)	<0.001	0.60	1.02 (1.01-1.03)	0.004	0.72
Women ≥ 65 years	869 (33.42)^g^	1.03 (1.03-1.04)	<0.001	0.62	1.02 (1.02-1.03)	<0.001	0.68

^a^OR: odds ratio; CI: confidence interval; All statistics measured a change of 0.01 on the frailty index.

^b^AUC: areas under the receiver operating characteristic curves.

^c^Analyses adjusted for age, BMI, smoking, drinking, and education.

^d^Defined as women had any falls during the follow-up but without baseline falls.

^e^Defined as women had any falls during the follow-up and with baseline falls.

^f^For any falls during the third year of follow-up; Analyses adjusted for age, BMI, smoking, drinking, education, and baseline falls.

^g^Numbers obtained after 10 multiple imputation.

Bootstrap analyses with replacement during the third year of follow-up were performed to assess the internal validation of the FI’s relationship to falls. Results for an increase of 0.01 on the FI remained unchanged (OR: 1.02, 95% CI: 1.02-1.03 for all the women; OR: 1.02, 95% CI: 1.01-1.03 for younger women; OR: 1.03, 95% CI: 1.02-1.04 for older women), indicating the relationship between the FI and falls was internally validated.

### Relationship between the FI and fractures, death and overnight hospitalizations

During the 3-year follow-up, 6.36% (n = 238) reported incident fractures (Table 3). The increment of 0.01 on the FI was related to increased risk of all ten fractures (i.e., clavicle, upper arm, wrist, spine, rib, hip, pelvis, ankle, upper leg and lower leg), with a hazard ratio [HR] of 1.02 (95% CI: 1.01-1.03) in the fully-adjusted model. The HR was 1.17 (95% CI: 1.09-1.25) measured by per 1-SD increment of the FI. Eighteen hip fractures were identified during the follow-up, and they were marginally associated with the FI (HR: 1.04, 95% CI: 1.00-1.07, P = 0.051 for an increase of 0.01 on the FI). There were 28 spine fractures and 208 other fractures (i.e., the other eight nonhip and nonspine fractures) reported, with a significant HR of 1.03 and 1.02 measured by per change of 0.01 on the FI respectively. No violations of the proportional hazards assumption were observed.

One hundred and seven women (2.69%) died during the follow-up (Table 3). The FI was significantly related with risk of death (OR: 1.05, 95% CI: 1.03-1.06 for an increase of 0.01 on the FI; OR: 1.40, 95% CI: 1.25-1.58 for an increase of per SD) in the fully-adjusted model, with an AUC of 0.80 (95% CI: 0.76-0.85). There were 8.75% (n = 347) women during the third year of follow-up reporting that they spent at least one night in hospital in the past 12 months (Table 3). When overall nights stayed in hospital were counted, a significant association was found in the fully-adjusted model between hospitalizations and the FI (incidence rate ratio [IRR]: 1.02, 95% CI: 1.02-1.03 for an increase of 0.01 on the FI; IRR: 1.18, 95% CI: 1.13-1.24 for an increase of per SD).

The results of age-adjusted models and subgroup analyses stratified by age-stratum were similar to those based on the fully-adjusted models for fractures, death and overnight hospitalizations (Table 3).

## Discussion

### Main findings

With the use of the GLOW Hamilton cohort, we constructed a FI of deficit accumulation and investigated its relationship to risk of falls, fractures, death and overnight hospitalizations. The FI was associated significantly with increased risk of falls. Results of subgroup and sensitivity analyses indicated the relationship between the FI and risk of falls was robust, while bootstrap analysis judged its internal validation. Positive associations were also identified between the FI and risk of fractures, death and overnight hospitalizations.

The underlying premise of the theory of deficit accumulation is that individuals with more deficits are more likely to be frail, which could be quantifiable using a FI [1]. Although each individual deficit may not carry an imminent threat of adverse events, the cumulative deficits contribute to the increased risk [1,2,28]. As recommended [3,10,29-31], the FI with 34 deficit variables covered multidimensional domains including medical conditions, ADL, symptoms and signs, and healthcare utilization. The consensus was reached by clinical judgement and discussion before each variable was included in the FI, thereby supporting the content validity of the FI [32]. Face validity was assessed and corroborated by the relationship between individual variable included in the FI and falls during the third year of follow-up (Table 2). The FI predicted the outcomes significantly and discriminated falls and death with acceptable AUCs (Table 3), which supported the predictive validity of the FI [32].

The FI was significantly correlated with chronological age. The estimated FI increased across ages per year with a similar rate to other studies (β = 0.02-0.03 on a log scale) [1,19,20]. Women < 65 years accumulated deficits to a lesser extent than older women, and the older women tended to be frailer and had more deficits prior to death [33]. The sub-maximal, age-invariant 99% upper limit to the FI was around 0.60, indicating that death would be inevitable when frail people accumulated deficits after that limit [19,34].

The incremental FI significantly predicted increased risk of falls, fractures, death and overnight hospitalizations. This implies that the FI would be helpful to plan and assess future interventions aiming at improving the health outcomes of the elderly. Of note, the risk of death increased with the incremental FI significantly, with the OR of 1.05 measured by per change of 0.01 on the FI (Table 3). This suggests the FI would be very meaningful to targeting people at high risk of death. For falls, results of sensitivity analyses showed that the FI was related to higher risk of recurrence than new incident falls (Table 4), after stratifying by baseline falls which was a significant risk factor for future falls [35,36]. Women with baseline falls were frailer (greater FI) and more likely to suffer recurrence triggered by minor stressor events [2], which therefore indicates that interventions to prevent future falls may be directed to the elderly people with previous falls.

Some types of fractures were likely due to trauma, thus being irrelevant to frailty [37]. However, significant associations persisted after subgroup analyses were performed, in which the relationship was stronger for hip and spine fractures than the other fractures (Table 3).

### Comparison with other studies

Compared with the phenotypic model [14-17,38-41], even though the FI approach to measure frailty has been less widely used [21], a FI could predict risk of adverse outcomes more precisely than a phenotypic model did [8,42]. However, current evidence of the relationship between FI and falls, fractures, death and overnight hospitalizations is sparse.

Our results agreed with the findings from a Chinese study which used the FI with 33 health deficits to examine risk of health adverse outcomes in community-dwellers aged 55 years and older [24], especially on the associations between recurrent/new incident falls and the FI during follow-up. Another previous study measured the increased risk of death with an increment of 0.01 on the FI (HR = 1.04, 95% CI: 1.04-1.05) in the elderly aged 70 years and older [19], which also yielded similar results to our findings (OR = 1.05, 95% CI: 1.03-1.06) (Table 3).

However, unlike other studies, we did not choose cutpoints to trichotomize participants into three groups (e.g., robust, prefrail and frail) [8,21,42,43], or categorize them as four groups based on the quartiles of the FI’s distribution [44]. Taking into account the diverse items included in the FI and the different populations used, the cutpoints would be heavily data-dependent and only statistically sensible, thus limiting the generalizability and clinical sense of the FI. For instance, a recent study using the whole GLOW cohort applied the phenotypic model to measuring frailty, in which it trichotomized women into robust, prefrail or frail group according to the scores of the components of frailty including slowness and weakness, poor endurance and exhaustion, physical activity, and unintentional weight loss [17]. If we chose the cutpoints using Rockwood’s methodology [42] by overlapping the FI’s density distributions of the robust, prefrail and frail women, our cutpoints would be 0.20 and 0.35 corresponding to the crossing points of the three groups. These cutpoints would be quite distinct from what they selected as 0.08, 0.25, respectively [21,42], such that results were not comparable and analyses using these cutpoints were meaningful merely statistically rather than clinically.

### Strengths and limitations

Using a FI to quantify the change in the health status of the elderly would be a major concern to geriatricians and to population planners [19]. Our study used data from the GLOW Hamilton cohort to construct a FI of deficit accumulation, and investigated the FI’s validity, discriminability and prediction systematically. Other strengths of this study include the prospective design, the large sample size and the representative sample given the unique sampling method [18]. Non-selected women over a broad age range were recruited based on the lists provided by their physician practices with few exclusion criteria, so that the overall participants should be representative of the practices [45,46]. Our FI can be considered for prediction of risk of falls, fractures, death and overnight hospitalizations. However, external validation of these findings along with comparison with other existed assessment tools such as Garvan Fracture Risk Calculator [47], Fracture Risk Assessment Tool (FRAX) [48] and Fall Risk Assessment Tool (FRAT) [49], are needed in the future research.

However, our data must be interpreted with caution. The data was collected by patient self-report and could not be validated by medical records. Nevertheless, self-reported data have been shown to be reasonably credible for health adverse outcomes in different populations and settings [50-54]. There was also unquantifiable recall bias when participants were answering questionnaires. However, it improves efficiency and methodological consistency, aids in the data collection from a large sample, and maximizes the power for a relatively infrequent health adverse outcomes [55]. Besides, the population in the GLOW was only composed of women, and therefore our results may not be generalizable to elderly males. Moreover, even if it may not be difficult to use a FI of many items (n = 34 in our study) in geriatric medicine [21], the FI still necessitates additional clinical translation [42].

## Conclusion

To conclude, in this study the FI of deficit accumulation increased with chronological age significantly. The FI was related to and predicted increased risk of falls, fractures, death and overnight hospitalizations significantly. A validated and pragmatic FI would be considerably helpful for decision-making at a clinical research and health care policy level.

## Abbreviations

FI: Frailty index; GLOW: Global Longitudinal Study of Osteoporosis in Women; ADL: Activities of daily living; SD: Standard deviation; ANOVA: Analysis of variance; BMI: Body mass index; ROC: Receiver operating characteristic; AUC: Area under the curve; CI: Confidence interval; OR: Odds ratio; HR: Hazard ratio; IRR: Incidence rate ratio.

## Competing interests

The authors declare that they have no competing interests.

## Authors’ contributions

GL was responsible for the study conception and design, data analyses and the draft of manuscript. JDA enrolled participants, conceived the plan, and provided comments on the manuscript and data. GI, LP, CK, AP and LT made critical revisions to the manuscript, and provided professional and statistical support. All authors approved the final manuscript.

## Pre-publication history

The pre-publication history for this paper can be accessed here:

http://www.biomedcentral.com/1471-2474/15/185/prepub
